# Families’ Perception of Classic Ketogenic Diet Management in Acute Medical Conditions: A Web-Based Survey

**DOI:** 10.3390/nu12102920

**Published:** 2020-09-24

**Authors:** Ludovica Pasca, Costanza Varesio, Cinzia Ferraris, Monica Guglielmetti, Claudia Trentani, Anna Tagliabue, Pierangelo Veggiotti, Valentina De Giorgis

**Affiliations:** 1 Department of Child Neurology and Psychiatry, IRCCS Mondino Foundation, 27100 Pavia, Italy; valentina.degiorgis@mondino.it; 2 Department of Brain and Behaviour Neuroscience, University of Pavia, 27100 Pavia, Italy; 3 Human Nutrition and Eating Disorder Research Center, Department of Public Health, Experimental and Forensic Medicine, University of Pavia, 27100 Pavia, Italy; cinzia.ferraris@unipv.it (C.F.); monica.guglielmetti@unipv.it (M.G.); claudia.trentani@unipv.it (C.T.); anna.tagliabue@unipv.it (A.T.); 4 Department of Biomedical and Clinical Sciences, L. Sacco, University of Milan, 20157 Milan, Italy; pierangelo.veggiotti@unimi.it; 5 Department of Child Neurology, V. Buzzi Children’s Hospital, University of Milan, 20154 Milan, Italy

**Keywords:** drug-resistant epilepsy, ketogenic diet, families’ perception of care, emergency, web-based survey

## Abstract

**Objective:** To describe families’ experiences in managing epileptic patients undergoing ketogenic dietary therapies (KDTs) in acute medical settings. **Methods:** We conducted a short online survey addressed to the families of patients undergoing a classic ketogenic diet (cKD) for at least three months. The survey was composed of 18 questions exploring the following issues: demographic characteristics, epilepsy diagnosis, ketogenic-diet treatment history, the reason for emergency-ward admission and patient management, surgery-procedure management, and outcomes. **Results:** A sample of 50 families agreed to participate. Out of 50 patients, 33 (66%) had been undergoing a cKD for more than two years. Fifteen (30%) patients had been admitted at least once to the Emergency Room (ER), and 8.2% had undergone surgical procedures during cKD treatment. The causes of ER admission were the following: seizures, infection, trauma, and gastrointestinal or respiratory problems. In 75% of cases, blood ketonemia was not monitored during ER admission, and according to 46% of responders, the medical staff intervening did not have a basic knowledge of KDTs. **Conclusions:** According to both our experience and caregivers’ reports, it might be useful to search for standardized specific approaches to patients undergoing KDTs in the emergency setting.

## 1. Introduction

Ketogenic dietary therapies (KDTs) represent an established and effective treatment option for childhood drug-resistant epilepsy (DRE) [1], which is defined as insufficient seizure control after adequate trials of two tolerated, appropriately selected antiepileptic drugs [2] and affects about 10% to 30% of epileptic patients [3]. Moreover, KDTs are the mainstay of treatment for glucose transporter type 1 deficiency syndrome (GLUT1-DS, OMIM 606777), a rare metabolic encephalopathy [4] characterized by developmental impairment, epilepsy, and a movement disorder. The most frequently used ketogenic dietary therapy for epilepsy and GLUT1-DS is the classic ketogenic diet (cKD), a high-fat, very low carbohydrate, adequate-protein diet with a high ketogenic ratio (fat to carbohydrates + protein by weight) [5]. The cKD (with a 3:1 or 4:1 fat-to-carbohydrate-plus-protein ratio), which has been used to treat DRE since 1921, with minor changes in the last decades, triggers a systemic shift from glucose metabolism toward fatty acid metabolism, yielding ketone bodies as a substrate for brain energy [6]. The most recent international consensus guidelines for the management of children on KDTs was published in 2018 [7], focusing on updated clinical and research evidence for patient selection, pre-ketogenic diet evaluation, KDT introduction, and monitoring for potential side effects. Available efficacy data confirm that KDTs can be maintained with good compliance in pediatric and adolescent patients [1,8]. In particular, in a recent review by Schwantje and colleagues [9], it has been demonstrated that compliance with a ketogenic diet (KD) is higher than 80% in the GLUT1DS population. The conditions turn out to be more complicated for DRE patients. In this specific subset of patients, indeed, compliance appears to be high when the KD is administered through a gastro-intestinal tube [10]; nevertheless, in a recent review [1], drop out was reported at rates ranging from 10% at three months to 46% at 16 months, due to a lack of compliance and intolerability caused by adverse events.

However, with an individualized treatment and comprehensive prevention, the rate of side effects related to KDTs has been significantly reduced [1]. Nevertheless, patients undergoing KDTs present significant functional limitations and associated comorbidities, potentially leading to frequent admissions to the emergency department or extra-neurological wards, where medical staff might not be aware of how to cope with KDTs. Additionally, KDTs themselves, especially in certain conditions such as infections, could lead to complications requiring medical attention. There are insufficient data on specific recommendations and approaches to be adopted in the Emergency Room (ER) and in surgical and anesthesiological settings for patients undergoing KDTs.

We set up an online survey submitted to patients’ relatives aiming to assess caregivers’ perceptions about the management of a patient undergoing the cKD in the acute hospital setting.

## 2. Materials and Methods

An anonymous online survey was proposed to 54 families of pediatric epileptic patients undergoing cKD for at least three months and regularly followed up at the IRCCS C. Mondino Neurological Institute of Pavia and V. Buzzi Children’s Hospital of Milan, after approval from our Ethical Committee (P-20190033749). Fifty out of 54 families agreed to participate. The patients included had a diagnosis of GLUT1 deficiency syndrome or drug-resistant epilepsy of genetic or structural etiology. The survey items focused on symptom areas and treatment priorities. In the first section of the survey, participants were asked to report some information related to demographics, history of the disease, and KD treatment. The second section included items assessing the occurrence of ER admission and surgery procedures and parents’ perceptions of patient management in light of the KDTs.

The complete survey proposed is shown in Table 1.

## 3. Results

The majority of the patients included (66%) had been undergoing cKD treatment for more than two years at the time of survey completion, and most of them (52%) had an age ranging from two to eight years (see Table 1). Fifteen (30%) patients were admitted at least once to the ER, and 8.2% had undergone surgical procedures during KD treatment. The causes of ER admission were the following: seizures, infection, trauma, and gastrointestinal or respiratory problems. According to the answers provided, in most cases (90%), the medical staff who intervened took into consideration that the patient was undergoing a cKD, and contact with the referring epileptologist/keto-team became possible on the same day of hospital admission. In 75% of cases, blood ketonemia was not monitored during ER admission, and according to 46% of families, the medical staff who intervened did not have a basic knowledge of KDTs.

In those undertaking surgical procedures (8.6%), no pre- or post-procedural specific measures were adopted considering KD treatment.

In 36.7% of cases, the family had a list of replacement drugs that were glucose-free, provided by the Italian Association of GLUT1-DS, available for health care staff to apply in an acute setting. In another 30% of cases, the families declared contacting the referring keto-team for such information; in 18% of cases, the families referred to the general pediatrician, and in the remaining cases, the families declared themselves to be autonomous in such management.

Almost all the participants (98%) agreed with the proposal of distributing a shared protocol for medical staff and families for coping with urgent medical conditions considering the ongoing KDT treatment.

## 4. Discussion

The survey presented was realized to explore parents’ perceptions and expectations in the emergency setting in relation to the ongoing non-pharmacological KDTs, which have specific metabolic and clinical management implications.

Participation in the survey was satisfactory, since 50 families out of 54 agreed to fill in the anonymous online questions. Such a high response rate (almost 93%) was outstanding, especially considering this was an online questionnaire, for which response rates are generally lower than for postal or telephone questionnaires [11]. In our opinion, this datum might reflect the consistency of families’ concerns on this specific topic. As a referring keto-team (composed of a child neurologist, a nutritionist, and dietitians), we are always available by telephone and e-mail contact [12], thus assuring open access to communication even beyond scheduled visits and necessary exams. Moreover, we often interact with patients’ general pediatricians or other specialists in light of the ongoing KDTs and possible drug interactions or specific clinical monitoring in particular situations. In the emergency setting, it might not always be feasible to promptly interact with the family or the medical staff supposed to face the acute medical need. Furthermore, even if substantially improved compared to that in the past, the current knowledge on KDTs is not adequately shared in extra-neurological settings, thus often leaving the caregivers to provide main necessary details on KDT treatment to the medical staff.

Thirty percent of the patients analyzed were admitted at least once to the ER with ongoing KDTs. Similarly to in the report of Çağlar et al. [13], the reason for admission was often seizure- or infection-related, rather than an adverse effect of the KDTs. Gastrointestinal problems were also common in our population, and in the study of Çağlar et al. [13], vomiting was the most common complaint in ER visits. In the study mentioned above, the management of patients undergoing KDTs admitted to the ER was not explored. However, from the same study, it emerged that dextrose-containing fluids were administered mistakenly to nearly half of the patients [13].

From our survey, it emerged that in 75% of cases, ketonemia was not monitored during ER admission, and according to 46% of responders, the medical staff did not have the skills to manage cKD-related issues. Ketonemia represents a critical parameter for being registered to monitor cKD function and even other biochemical and clinical data. In the great majority of cases (90%), the medical staff who intervened took into consideration that the patient was undergoing cKD, and contact with the referring epileptologist/keto-team was possible at hospital admission. This rapid information exchange could partially fill the knowledge gap for KDTs when present. However, the risk of selecting the wrong treatment or data misinterpretation might occur.

As good practice, families should carry with them informative material on KDT treatment if available, to be given to the medical staff of the ER if needed.

In the few cases who underwent surgery, no post-procedural complications occurred, and every specific precaution was adopted before surgery in light of KDTs. However, the fasting duration, support therapies, and feeding after a potential gastrointestinal surgical procedure should be considered as unsolved themes to be addressed by experts. Similarly, data on anesthesiologic procedures are lacking, but there is evidence of awareness of the topic’s importance. In a recent case report on total intravenous anesthesia in a GLUT1DS patient [14], the authors consider the potential influence on the patient’s blood glucose level of different anesthetics and recommend normoglycemia during the perioperative phase and the avoidance of glucose-based medication to maintain the patient’s ketotic state.

Since parents’ priorities depend on the effectiveness of communication with physicians, in the acute setting, the information exchange could be suboptimal. The perception that the medical staff might not have sufficient skills in light of KDT management could be further destabilizing for caregivers. As underlined by Ammentorp et al. [15], the most critical outcome of pediatric care is the improvement of the child’s health or reduction of symptoms, and parents’ satisfaction is associated with such central outcomes, including adherence to the therapeutic regimen and understanding of medical information. Furthermore, parents’ satisfaction with care can be considered a useful proxy variable for some crucial aspects of care [16]. Therefore, identifying parents’ perceptions and expectations may represent an important insight for assessing care quality and physicians’ ability to provide treatment.

This study has some limitations. First, it considered a relatively small population of patients followed in a tertiary-level center; secondly, it relied on parents’ reports, and thus patients’ and physicians’ perspectives were not investigated. Then, clinical data about KD compliance, effectiveness, and side effects were not extensively analyzed because this was not the purpose of the study and because parents’ reports are a partial source of this type of information.

**Figure 1:**
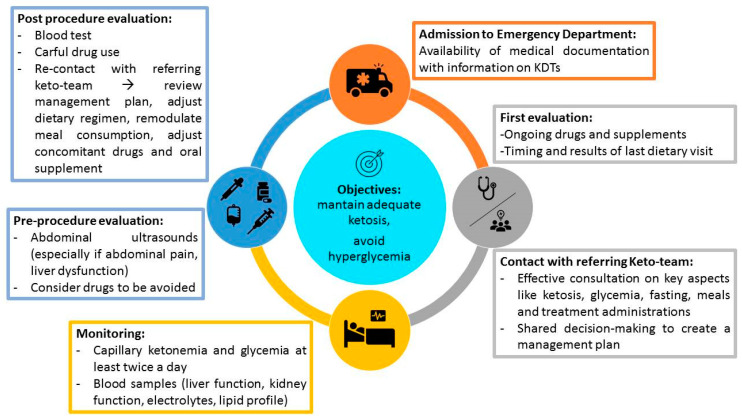
This figure shows indications and concerns for practitioners dealing with children on a ketogenic diet (KD) in emergency settings.

Following the evidence outlined by this survey, a consensus statement providing specific indications for KDT management in the acute setting to share among different countries where KDTs are utilized should be an essential tool to achieve and be offered to families and emergency doctors. In particular, in the event of an emergency requiring access to the ER, we propose families and patients should always carry a type of “vademecum” represented by a summary of their clinical history with dietary indications, therapies, and supplement prescriptions (an example is available on the GLUT1DS Italian Onlus association website www.glut1.it). At the time of first evaluation, we suggest that ER referring physicians should promptly contact the patient’s referring keto-team to share a management plan. In our opinion, particular attention should be paid to critical aspects such as ketosis, glycemia, fasting, meals, infusions and treatment administrations. While staying in ER, we suggest the careful monitoring of blood tests and capillary ketonemia and glycemia evaluation at least twice a day. Whether medical or surgical intervention is required, we suggest a careful and cautious choice of drugs to be administered considering their potential effects on ketonemia and glycemia. Once medical or surgical procedures are completed, referring ER physicians should recontact the patient’s referring keto-team to review the management plan, adjust the dietary regimen, remodulate meal consumption, and adjust concomitant drugs and oral supplements. For a proposed framework on emergency management in patients undergoing a KD, see Figure 1.

## 5. Conclusions

The aim of the survey presented was to estimate the importance of improving the care of patients undergoing KDTs in an acute medical setting, to delineate therapeutic approaches needed in a better way. We believe that future guidelines should focus on general patient management in the acute setting, providing specific indications on ketonemia monitoring, fasting and refeeding procedures, and drug administration.

## Figures and Tables

**Table 1:** Web-based survey.

1. How old is your child?	2–8 years: 52% 9–14 years: 26% >14 years: 22%
2. Which is the diagnosis of your child?	GLUT1DS: 60% Drug resistant epilepsy of genetic origin: 25% Drug resistant epilepsy of structural etiology: 15%
3. How long has your child been on KD?	More than 2 years: 66% 1 year: 22% From 3 months to 12 months: 12%
4. Does your child have a percutaneous endoscopic gastrostomy?	Yes: 2% No: 98%
5. Does your child utilize a pre-generated ketogenic formula?	Yes: 44.9% No: 55.1%
6. Which side effects attributable to KD did your child manifest?	GI side effects:15% Renal stones: 2% Liver problems: 2% Blood test alterations: 7% Inappetence: 5% Growth retardation: 16% None: 53%
7. When undergoing KD, did your child need an admission to the ER?	Yes: 30% No: 70%
8. Which was the reason why your child was taken to the ER?	Uncontrolled seizures/status epilepticus: 42% Infection: 23% Trauma: 5% Side effects due to KD: 15% Respiratory problems: 15%
9. Did the medical staff who intervened in the ER take into consideration that your child was undergoing KD?	Yes: 35.7% No: 74.3%
10. Was ketonemia monitored during the admission to the ER?	Yes: 25% No: 75%
11. Did your child undergo surgical procedures during KD treatment?	Yes: 8.2% No: 91.8%
12. Did the medical staff who intervened in the ER/surgery ward show a basic knowledge of KD?	Yes: 54% No: 46%
13. Did the medical staff who intervened in the ER askfor a phone contact with the referring centre/general doctor to share the indications regarding KD treatment?	Yes: 44.3% No: 55.7 %
14. Whenever a surgical procedure has been scheduled, were there pre-or post- surgical complications?	Yes: 0% No: 100%
15. In light of the scheduled surgical procedure and the ongoing KD treatment, were there specific precautions provided?	Yes: 0% No: 100%
16. Did you inform the referring epileptologist with regard to the acute medical situation which occurred?	Yes, the same day of ER admission: 74.3% Yes, a few days after the ER admission: 8.6% Yes, at the subsequent follow-up visit: 8.6% No: 8.6%
17. Do you have a list of replacement drugs (without glucose) to be carried with you or do you ask for information to the referring doctor?	We manage it independently: 14.3% We utilize a list provided by GLUT1DS family association: 36.7% We usually refer to the general pediatrician: 18.4% We usually refer to the keto-team: 30.6%
18. Do you think it could be useful to share with patients and families and medical specialists updated guidelines on KD management in the acute setting?	Yes: 94% No: 6%

## Abbreviations

KDTs	Ketogenic dietary therapies
DRE	drug-resistant epilepsy
KD	Ketogenic diet
cKD	Classic ketogenic diet
DRE	Drug-resistant epilepsy
GLUT1DS	GLUT1 transporter deficiency syndrome
ER	Emergency room
